# Deep imitation learning for 3D navigation tasks

**DOI:** 10.1007/s00521-017-3241-z

**Published:** 2017-12-04

**Authors:** Ahmed Hussein, Eyad Elyan, Mohamed Medhat Gaber, Chrisina Jayne

**Affiliations:** 1 School of Computing Science and Digital Media, Robert Gordon University, The Sir Ian Wood Building, Garthdee Rd, Aberdeen, AB10 7GE UK; 2 School of Computing and Digital Technology, Birmingham City University, 15 Bartholomew Row, Birmingham, B5 5JU UK

**Keywords:** Deep learning, Convolutional neural networks, Learning from demonstrations, Reinforcement learning, Active learning, 3D navigation, Benchmarking

## Abstract

Deep learning techniques have shown success in learning from raw high-dimensional data in various applications. While deep reinforcement learning is recently gaining popularity as a method to train intelligent agents, utilizing deep learning in imitation learning has been scarcely explored. Imitation learning can be an efficient method to teach intelligent agents by providing a set of demonstrations to learn from. However, generalizing to situations that are not represented in the demonstrations can be challenging, especially in 3D environments. In this paper, we propose a deep imitation learning method to learn navigation tasks from demonstrations in a 3D environment. The supervised policy is refined using active learning in order to generalize to unseen situations. This approach is compared to two popular deep reinforcement learning techniques: deep-Q-networks and Asynchronous actor-critic (A3C). The proposed method as well as the reinforcement learning methods employ deep convolutional neural networks and learn directly from raw visual input. Methods for combining learning from demonstrations and experience are also investigated. This combination aims to join the generalization ability of learning by experience with the efficiency of learning by imitation. The proposed methods are evaluated on 4 navigation tasks in a 3D simulated environment. Navigation tasks are a typical problem that is relevant to many real applications. They pose the challenge of requiring demonstrations of long trajectories to reach the target and only providing delayed rewards (usually terminal) to the agent. The experiments show that the proposed method can successfully learn navigation tasks from raw visual input while learning from experience methods fail to learn an effective policy. Moreover, it is shown that active learning can significantly improve the performance of the initially learned policy using a small number of active samples.

## Introduction

Recent years have seen a rise in demand for intelligent agents capable of performing complex motor actions. Advances in robotics and computational capabilities provide opportunities for many potential applications such as assistive robots, autonomous vehicles and human computer interaction. However, the challenge remains to create intelligent agents capable of robust and effective behavior. Most applications are dynamic and involve many variables and are therefore not suitable for manually designed policies. It is also difficult to breakdown and articulate how humans perform tasks in order to program intelligent agents to replicate this behavior. For instance, it is hard for an experienced driver to describe to another human how to drive well. A more intuitive and effective method of imparting this knowledge is to show the student examples of good driving.

Imitation learning is a paradigm where an intelligent agent is taught to mimic human behavior by supplying the agent with demonstrations provided by a teacher rather than instructions. By learning from demonstration, the agent does not require explicit knowledge about the task or the environment such as objectives or constraints. Instead a generic learning process is advocated where all needed information is inferred from the provided demonstrations. Two major challenges facing imitation learning are (1) creating adequate feature representations for learning. (2) Learning a policy that generalizes to unseen situations. Feature representations are required to encode the demonstrations in a way that the agent can learn from and also to represent how the agent perceives its environment from its sensory data. The representations must be adequate for learning as well as be suitable for real-time processing. Manually designing suitable features for imitation learning is an arduous task as different representations must be tailored for each task or environment, especially in dynamic settings where the representations must be robust against various scenarios. Generalization to unseen scenarios is also a challenge because of the dynamic nature of the tasks. This is a common problem because demonstrations typically show the best way to perform a task and do not offer any information about recovering from sub-optimal actions. Therefore, approaches are required that can generalize beyond demonstrated behavior without extensive feedback from a teacher or the environment. This paper builds on the work reported in [1] and presents a deep active learning method for learning from demonstrations in navigation tasks. The proposed method addresses the challenges of imitation learning by utilizing deep learning to learn feature representations and active learning to improve generalization using a relatively small number of samples. The main extension in this paper is comparing the proposed methods with state-of-the-art deep reinforcement learning methods as well as creating methods for combining reinforcement learning with learning from demonstrations. Utilizing both taught behavior and experience in learning aims to mitigate the limitations of each approach. By allowing the agent to explore using trial and error, it is exposed to new scenarios and is able to generalize without requiring a teacher’s involvement. While demonstrations can provide a starting point to learn more efficiently than learning from scratch using trial and error.

The proposed learning method is generic and does not require any prior knowledge of the task. The only information presented to the agent is the demonstrations, which are acquired by controlling the agent using a deterministic optimal policy. For each frame, the agent’s point of view and the action performed are captured and used to construct a dataset of observation/action pairs. A deep convolutional neural network is trained on the captured dataset to learn a policy that mimics the demonstrated behavior. Since direct imitation can lead to poor generalization, active learning is employed to adapt to situations that are not represented in the demonstrations. Active samples are selected based on the confidence of the agent’s current policy. The agent queries the optimal policy to suggest actions for these instances. The trained policy interacts with the 3D environment in real time, observing the current state, extracting features and predicting the action to perform in a timely manner. The proposed learning from demonstration method is compared to two popular deep reinforcement learning methods: deep-Q-networks (DQN) which has shown human level behavior on learning Atari games from raw pixels and paved the road for deep reinforcement learning methods, and Asynchronous actor-critic (A3C) learning that is considered the state of the art in deep reinforcement learning and has shown success on a 3D navigation task. Moreover, we investigate methods for combining learning from demonstrations and reinforcement learning to alleviate the generalization limitations of imitation methods and help reduce the search space of trial and error methods. Extensive experiments are conducted on four navigation tasks in the 3D MASH simulator [2] as well as a simple 2D navigation task to analyze the performance of the methods used in this paper. The evaluation highlights the challenges and advantages of the different approaches.

In the next section we provide a background to reinforcement and imitation learning methods and highlight our motivation. Section 3 reviews related work in the literature. Section 4 describes the proposed methods. Section 5 details the conducted experiments and results. The paper is concluded in Sect. 6, and future steps are discussed.

## Background

Deep learning methods have shown great success in learning from high-dimensional raw data in a variety of applications. Convolutional neural networks (CNN) are used in many computer vision applications to learn from raw pixels and achieve state-of-the-art results in various image classification tasks [3, 4]. CNNs are effective because they employ multiple convolution layers that automatically extract higher level patterns from the input features which are more useful for learning. Automatically extracting feature representations can greatly facilitate creating generic learning processes for learning from demonstration. Where the same network architecture can extract relevant features for different situations depending on the provided demonstrations.

A different approach for creating intelligent behavior in agents is learning from experience. Learning from experience relies on trial and error and uses reinforcement learning to train a policy based on feedback from a reward function. Deep reinforcement learning is rapidly gaining attention due to recent successes in a variety of problems [5–10]. The combination of deep learning and reinforcement learning allows for a generic learning process that does not consider specific knowledge of the task and learns from raw data. Reinforcement learning (RL) is a popular choice for learning motor actions because most tasks can be modeled as a Markov decision process. Moreover, optimizing a reward function arguably provides a better description of a task than optimizing a policy [11]. Learning from experience can produce robust policies that generalize to dynamic scenarios by balancing exploration and exploitation of rewards. However, finding a solution through trial and error may take too long, especially in problems that require performing long trajectories of actions with delayed rewards. In such cases it may be extremely difficult to stumble upon rewards by chance. And the time to learn a policy to maximize the rewards exponentially increases. Such challenges are present in many real-life applications and pose limitations to current methods. Another drawback is that learning through trial and error may result in a policy that solves the problem differently to how a human would. Performing a task in a manner that is intuitive to a human observer may be crucial in applications where humans and intelligent agents interact together in an environment.

On the other hand, learning from demonstrations may result in faster learning and produce a policy that follows the teacher’s way of solving the task [12]. However, learning a direct mapping between observation and action can commonly result in a policy that generalizes poorly to unseen scenarios. The supervised policy only learns to deal with situations covered in the demonstrations. Since demonstrations only cover the optimal trajectory, if the agent deviates even slightly from that trajectory at any point (which is expected in any machine learning application), it finds itself in an unseen situation not covered by the training data [13]. So essentially the policy is trained using samples from a distribution that is different to the one it is evaluated on. Therefore, in many cases, policies need to be refined based on the performance of the initially learned policy. Moreover, supervised learning needs a sufficient number of demonstrations which for deep network architectures may be large.

Navigation is an important skill for intelligent agents due to its relevancy to a variety of applications. Navigation can be a main task as in autonomous vehicle applications [14–20] or as a base skill for other tasks such as humanoid robots which need to move before performing other tasks [19, 21]. Navigation tasks present a set of problems where the agent is typically required to perform long trajectories and receives rewards at the end of the trajectory. In many applications, it is not realistic to design intermediate rewards and is common in navigation tasks to only provide terminal rewards after reaching the target. Navigation from visual input also poses an extra challenge as the view of the agent changes constantly as it moves around the environment making it more difficult to observe relations between subsequent states. This is in contrast for example to object manipulation tasks where a static view contains all the information needed by the agent, and changes from one frame to the next can be more easily tracked.

## Related work

In this section we present related work and review methods that utilize deep learning in imitation learning and reinforcement learning methods. This section also surveys different methods proposed in the literature to combine learning from demonstrations and experience.

### Navigation

From an early stage, artificial intelligence (AI) research has accorded special interest to navigation problems as many potential applications rely on autonomous navigation. Learning from demonstrations lends itself to navigation problems as it is difficult, even for experts, to identify an optimal strategy for agents to follow in complex environments. Prioritizing different aspects of navigation such as speed, safety and avoiding obstacles can be better inferred from demonstrations [11]. An early work [14] proposed a method for learning autonomous control of an aerial vehicle from demonstrations. Since then several papers have proposed learning autonomous aerial navigation using demonstrations [22] and reinforcement learning [15, 16, 23]. In [19], a robot learns how to navigate through a maze based on its sensory readings. The information available to the robot is a stream from an infrared (IR) sensor and input from a controller operated by a teacher. The agent learns to map its sensory data directly to the motor primitives provided by the controller. The IR data provide information about the proximity of objects. This sensory information does not allow the agent to differentiate between different objects. In [24], a laser sensor is utilized to enable the agent to detect and identify relevant objects. Instead of mapping the sensory data directly to motor primitives, the agent learns to identify sub-goals from its observations. A more detailed representation of the environment can be provided by visual data. High-dimensional visual data can be efficiently provided to intelligent agents thanks to advances in computational resources and communication technology. An agent learns to play a racing game from visual data in [25]. A teacher plays the game using a controller, and the controller’s input is captured along with the game’s video stream to create a training dataset. The video stream is stored as raw pixels, and down-sampled versions of the frames are input into a neural network. In [9], a deep reinforcement learning algorithm is used to teach an agent in a racing simulator from raw visual features. The learned policy maps the high-dimensional visual input to multiple continuous outputs such as steering and pressing the acceleration pedal. Another racing application is demonstrated in [26] where the training algorithm uses features extracted from the simulator (such as the position and speed of the car). It is shown that learning from demonstration can be used to handle high degree of freedom low level actions; however, features such as those extracted from the simulator are difficult to produce in real-world applications. Learning from visual information is not limited to the point of view of the agent. In [17], an imitation learning method is proposed to train a vehicle to navigate over long distances by learning from overhead data captured from satellite and aerial footage. Recently, state-of-the-art deep reinforcement learning methods have been evaluated on 3D navigation tasks [27, 28]. However, these benchmark tools are not publicly released.

### Deep learning from demonstrations and experience

Creating feature representations is one of the major challenges in developing intelligent agents; especially in dynamic environments. Engineering features that are robust in all situations facing the agent is very difficult. Therefore, deep learning methods are suitable for such tasks due to their ability to learn from raw sensory data. Recently, deep reinforcement learning methods have been gaining a lot of attention due to recent successes. One of the first successful deep reinforcement learning methods is deep-Q-networks (DQN) [5, 29] in which a convolutional neural network is used to estimate the *Q*-function from raw visual data. In order to scale *Q*-learning to a complex model such as CNNs, a replay buffer of training samples is collected from the performing policy and random mini-batches from the buffer are used to perform off-policy training. This buffer is important as it allows for random sampling of instances from different situations within the task. This technique has shown human level performance on several Atari games and paved the road for deep reinforcement learning methods. A similar concern is raised in [30] where a reservoir of liquid state machines (LSM)-based method is proposed to overcome over correlation between the training samples and the network’s sensitivity to the input. For a survey of reservoir-based methods refer to [31]. Since *Q*-functions provide an estimated reward for each possible action, *Q*-learning methods can only be applied to tasks with discrete actions. To use deep reinforcement learning in tasks with continuous action spaces, [9] adapts the contributions of DQN are adapted to an actor-critic reinforcement learning method. This algorithm consists of an acting step, in which a convolutional neural network outputs an action in continuous space, and a critic step where the rewards from the environment are used to evaluate the performed action. This approach is demonstrated to successfully learn tasks that require continuous input such as racing simulators from raw pixels. In [28], a number of asynchronous deep reinforcement learning methods are proposed. Instead of the replay buffer, these methods enforce diversity in the training samples by creating parallel threads in which multiple agents are acting; each in its own environment. Discarding the replay buffer and relying on parallel online learning allows both on-policy and off-policy reinforcement learning methods to be adapted to this approach. The best results from the methods proposed in this work belonged to Asynchronous advantage actor-critic (A3C), and set a new state of the art on the Atari benchmark and showed success on a 3D navigation task. A3C has been evaluated using a feed forward network similar to the one used in DQN and a long short-term-memory (LSTM) network that considers the past when predicting a new action. A version of A3C has been modified in [10] to take an image of the target as input in addition to the current view of the agent. The results show that this extra information significantly decreases the time required to reach the target.

Although most efforts focus on incorporating deep learning in reinforcement learning methods, examples of good behavior provided by an expert can significantly reduce the policy space and result in more efficient learning. If sufficient training samples are available, deep learning can be used to learn an effective policy from demonstrations. A drone is trained to navigate through cluttered environments in [32] using a dataset of good and bad examples (crashes). A camera mounted on the drone provides images of the environment in front of it. These images are used by a deep neural network to decide whether to move forward or not. If the drone does not move forwards it will turn to face a new direction and feed the new images to the network to make a decision. The deep network used for training follows the AlexNet architecture [4] and uses 2 output nodes to perform the binary classification. In [8], demonstrations for the Atari Benchmark used in [29] are generated using an offline Monte Carlo policy. These demonstrations are used to train a deep convolutional neural network in a supervised manner where the network predicts the likelihood of performing actions rather than expected rewards. The results show that the supervised policy learned from demonstrations outperforms DQN on Atari games. Similarly in [33], DQN is compared to learning from demonstrations on a game of “Pacman.” The demonstrations were provided by the authors playing through the game. The results show that the imitation learning approach resulted in an agent that can play the game effectively, while DQN failed to learn a well-performing policy. Most of the researches that utilize demonstrations with deep learning do so in combination with learning from experience to get the benefits of both approaches.

### Combining learning from demonstrations and experience

A common paradigm in combining learning from demonstrations and experience is to train the agent using reinforcement learning while using demonstrations to provide information that helps the reinforcement learning process. One such method is apprenticeship learning [11] where demonstrations are used to infer a reward function rather than to directly train a policy. Therefore, apprenticeship learning does not need to receive explicit rewards from the environment. Instead, it is assumed that the demonstrator is attempting to solve the task in a manner that optimizes an unknown reward function. The demonstrations are then used to learn an estimation of this reward function. The learned reward function provides feedback to the reinforcement learning algorithm in order to learn a policy. This approach in addition to not requiring an explicit reward system has the advantage of creating a policy that follows the demonstrator’s priorities. However, insufficient demonstrations that don’t cover possible scenarios can affect the generalization ability of the agent by creating an inadequate estimation reward function. Deep learning has been integrated with apprenticeship learning to train the reinforcement learning algorithm from raw pixels using a convolutional neural network [34].

In [6] supervised learning is used in two different ways to assist deep reinforcement learning to learn to play the board game “GO.” Firstly, a dataset of previous games is used to train a supervised convolutional neural network to play the game. The weights of the network are used to initialize the network used for reinforcement learning, so the agent starts exploring from a good starting policy. Secondly, a set of recorded games is used to train a network to predict whether the game will end in a win or a loss given the current state. This evaluation function provides feedback to the reinforcement learning algorithm so it can learn from the estimated consequences of each action. This method significantly outperforms direct imitation [35] and has shown the ability to beat human experts.

Guided policy search [36] allows combining learning from demonstrations with policy search reinforcement learning. A model-based approach generates guiding samples from demonstrations using differential dynamic programming (DDP). A model-free policy search algorithm then uses these sample trajectories to explore areas in which it is likely to be rewarded. By following the guidance of demonstrations the agent has faster access to rewards, which expedites learning through reinforcement learning. In [7], supervised and reinforcement learning are combined to perform deep end-to-end training on a number of object manipulation tasks. This approach does not require a dedicated teacher as the demonstrations are generated using a reinforcement learning policy. This policy is trained with knowledge of the positions of relevant objects. Generated trajectories of successful behavior are used to train a supervised convolutional neural network. The agent now learns the task from visual input with no information about the positions of objects. In [37], demonstration is used to initialize reinforcement policies. Because RL agents require a large number of trials before it achieves acceptable performance, using RL in many real-world applications may not be practical. Therefore, demonstrations are used to train an initial policy using supervised loss as well as temporal difference (TD) loss. DQN is then used to re-train the policy by continuing to optimize the TD loss. This method shows significantly faster learning than using DQN from scratch and outperforms using RL only on a number of Atari games.

### Active learning

Instead of using demonstrations to expedite reinforcement learning, a different approach would be to improve the generalization ability of supervised methods. This requires using the supervised policy’s performance to generate corrective feedback. In active learning, the agent is allowed to act according to its initially learned policy and queries the expert when in situations of low confidence. The expert provides the agent with the optimal action which enables it to continue exploring this previously unrepresented situation. These active samples are collected and used to re-train the policy, thus improving its weakest areas. In [19], active learning helps a robot to explore navigation tasks. With each action predicted by the robot’s policy, an estimate of the policies confidence in this action is calculated. In unfamiliar situations where the policies confidence is lower than a certain threshold, the robot queries a teacher for the correct action. These active samples help the robot explore unseen scenarios based on its initial policy and improve its ability to generalize. Due to the nature of imitation learning applications, it can be difficult for the teacher to provide active feedback when queried mid-trajectory. Therefore, in some applications the teacher can prompt the active corrections in contrast to traditional active learning. For example, in [38] the teacher identifies errors in the robot’s actions and physically corrects the robot’s movement during the performance. These adjustments are identified by the learner and used as active demonstrations. However, learner queries can still be employed to improve action trajectories. In [39], this problem is reduced to independent and identically distributed (IID) active learning and allows the agent to query the teacher at any step in the trajectory. Another special version of active learning can be seen in human–robot cooperation tasks. The robot and human are mutually dependent in their attempts to achieve a common goal. So as the human adapts to the robot’s action, the robot in return needs to adapt to the updated scenario. In [40], human–robot interaction occurs in rounds with an episode of active learning taking place between each round. The active learning stage updates the robot’s policy to accommodate for human behavior unseen in its initial training. While in the interaction round, the human modifies their behavior according to the robots actions. This process is repeated until the mutual actions of the interacting parties converge into a smooth cooperative behavior.

## Methods

This section presents the proposed method for learning from demonstration using active learning and deep neural networks. Methods for combining learning from demonstrations and experience using deep networks are also described.

### Deep active imitation learning

The proposed method is divided into three processes: (1) collecting demonstrations. (2) Supervised training of the neural network. (3) Active learning to refine the initially learned policy. This novel method combines supervised deep learning with data aggregation using active learning to produce a robust imitation learning approach with a relatively small number of training samples. Table 1 summarizes key differences between the proposed method, deep active imitation (DAI), and other approaches that use deep learning that learn from raw pixels, deep-Q-networks (DQN) [29] and deep guided policy (DGP) [7]. The table shows differences in the approaches such as the methods used to generalize the policy to unseen scenarios, the methods used to gather demonstrations and how the states are constituted from the captured frames. Moreover, it shows differences in the tasks and environments in which the different approaches are utilized. The viewpoint is the perspective from which the state of the environment is captured. Having a fixed point of view may help keep track of changes in the state, while having a dynamic viewpoint can be more challenging as the scene changes completely with small movements in the viewpoint. The trajectory refers to the sequence of steps typically needed to successfully complete the task. A longer trajectory can be harder to learn as small errors mid-trajectory can propagate and cause failure to reach the target. The environments refer to the settings in which the experiments are conducted. The environment can be randomized at every run, so the agent is faced with unfamiliar states. The more random the environment, the more the agent’s policy needs to generalize to the changing circumstances.

**Table 1:** A comparison of deep learning agent approaches

Method	DAI	DQN	DGP
Input	Pixels	Pixels	Pixels
Generalization	Active learning	*Q*-learning	Policy gradient
State representation	Greyscale frame	4 Greyscale frames	RGB frame
Demonstration source	Teacher	Reinforcement learning	N/A
Viewpoint	Dynamic	Static	Static
Trajectory	Long	Various	Shorter
Environment	3D simulator	2D simulator	Real world
Randomization	Extensive	Extensive	Limited

**Fig. 1:**
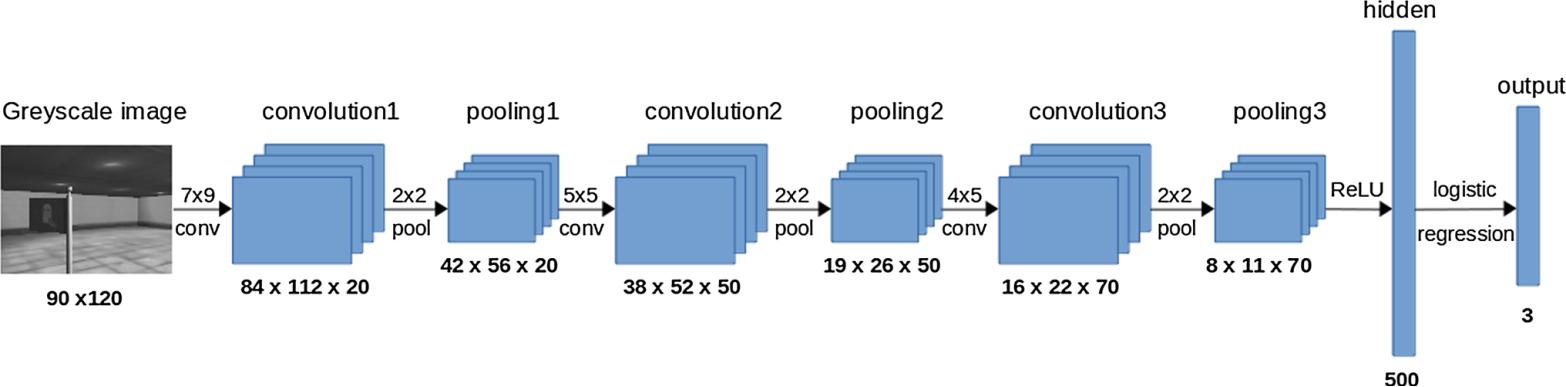
Architecture of the neural network used to train the agent

We begin by describing the process of collecting demonstrations. The demonstrations are collected from the point of view of the agent while being controlled by the teaching policy. A teacher providing demonstrations may be assumed to be optimizing an unknown optimal function. Therefore, as the teaching policy we use a deterministic optimal policy $\pi ^*$ to control the agent. This policy has access to information from the simulator such as the position of the agent and the target in 3D space in order to deterministically calculate the optimal action. For each frame *t* the view of the agent is captured as well as the action chosen by the optimal policy. This pair $(x_t,y_t)$ is added to the dataset of demonstrations $D=(x,y)$ where $x_i$ is a $120 \times 90$ image and $y_t$ is the action predicted by $\pi ^*(x_t)$. Only one frame is used in an instance $(x_t,y_t)$ as opposed to a sequence of consecutive frames which is usually used in deep reinforcement learning. Many AI applications are formulated as a Markov decision process (MDP) where the current state on its own is sufficient to predict the action to perform. And while deep reinforcement learning methods [5, 29] commonly represent the state by a sequence of frames, in the navigation tasks at hand the current view of the agent is enough for the optimal policy to make a decision. Next the captured dataset *D* is used to train the policy $\pi$ such that $u = \pi (x,\alpha )$. Where *x* is a $120 \times 90$ image and *u* is the action predicted by policy $\pi$ for input *x*, and $\alpha$ is the set of policy parameters that are changed through learning. The policy is learned using a deep convolutional neural network. The network used has 3 convolution layers with rectifier unit activation functions. Each layer automatically extracts higher level features from its input. The input to the first convolution layer is a luminance map of the captured $120 \times 90$ image. This transformation allows us to use one channel for greyscale instead of three channels for the RGB colors. Each convolutional layer is followed by a pooling layer to further reduce the dimensionality. Following is a fully connected layer with a rectifier unit activation function and finally an output layer which directly represents the action available to the agent. Figure 1 and Table 2 show the architecture of the network

**Table 2:** Neural network architecture

Layer	Size of activation volume
Input	$120 \times 90$
Conv1	$7 \times 9 \times 20$
Conv2	$5 \times 5 \times 50$
Conv3	$4 \times 5 \times 70$
FC	500
Output (FC)	3

Finally, active learning is used to adapt the initial policy learned from demonstrations to new situations that arise from the agent’s behavior. The agent is allowed to act in the environment according to its current policy. The agent’s confidence in its actions is estimated in order to identify weaknesses in the initial policy. In each frame the agent’s network provides a probability for each possible action. If probabilities to perform all actions are similar, then it is implied that the agent is not confident about which action to take in the current state. The opposite is the case if one action is far more probable than the rest. The confidence of the agent is estimated as the entropy of the action probabilities.

1 $$\begin{aligned} H(X)= - \sum \limits _{i}{P(x_i) \log _2 P(x_i)} \end{aligned}$$

where *X* is a vector representing the output of the final layer in the network, $P(x_i)$ is the probability of taking action *i*. The agent queries the optimal policy if its confidence is lower than a certain threshold. The action returned by the optimal policy and the current frame are recorded in a dataset of active samples. The active samples represent situations that were unseen in the initial training data but are likely to appear when executing the current policy. Thus, active learning helps the agent generalize to relevant scenarios. The training dataset is augmented with the active dataset and used to update the agent’s policy. The policy is updated by keeping the network weights learned during supervised learning when training the network using the augmented dataset. Initializing the weights in this way results in faster and easier convergence as retraining the network from scratch with the augmented dataset can be time consuming [41]. The training set used in this step includes both the active samples and the samples originally collected from demonstrations. If the network is only updated with the active samples, the initial policy is forgotten and replaced by one solely learned from the active samples, which is not sufficient [42]. This is known as the catastrophic forgetting phenomena [43] and can have severe effects on the agent’s performance if the network was trained online using the acquired active samples.

Algorithm 1 shows the proposed method.

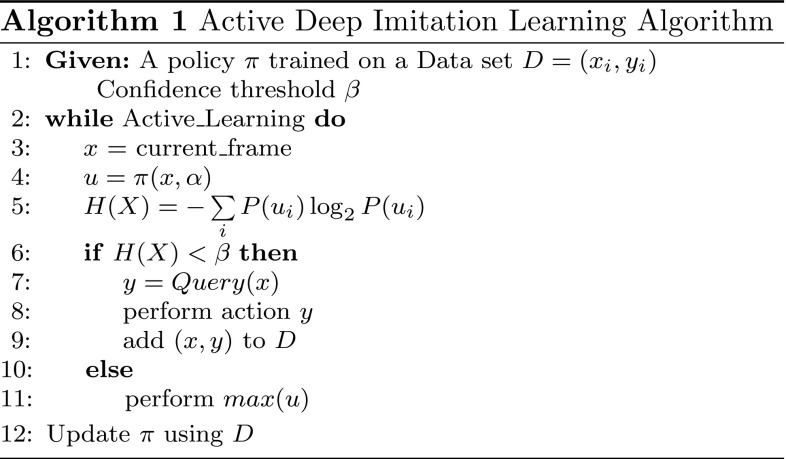



### Combining deep learning from demonstrations and experience

In this section we propose methods for combining learning from demonstrations and experience. The policy is learned using DQN [29] while using teacher demonstrations to expedite reinforcement learning. While a demonstrated instance is represented as a pair (*x*, *y*), in reinforcement learning additional attributes are added to represent an instance as a tuple $(s,a,r,s')$. *s* describes the current state of the agent in its environment and corresponds to *x* in demonstrations. *a* is the action taken by the agent and belongs to the same set of possible actions as *y*. *r* is a reward provided by the environment for performing action *a* in state *s* and $s'$ is the resulting new state. Reinforcement learning assumes the task takes place in an environment $\epsilon$. An experience is represented as a tuple $(s,a,r,s')$ where *s* is the state, *a* is the action taken at state *s*, *r* is the reward received for performing action *a*, and $s'$ is the new state resulting from that action. To combine learning from demonstrations and experience, the agent is trained using deep reinforcement learning, while demonstrations are used to facilitate the training process. The reinforcement learning algorithm follows [5] and uses a convolutional neural network to learn discounted rewards for performed actions. The network optimizes a *Q*-function *Q*(*s*, *a*) that predicts an estimated reward for the input state action pair. The *Q*-function is learned recursively using the Bellman equation.

2 $$\begin{aligned} Q(s,a) = E_{s' \epsilon }[r+\gamma {\mathrm{max}}_{a'} Q(s',a')|s,a] \end{aligned}$$

where $\gamma$ is a discount parameter and ${\mathrm{max}}_{a'} Q(s',a')$ is the largest estimated reward available to the agent at the next state $s'$. In the case where *s* is a terminal state which ends the task, $Q(s,a)=r$ as there is no future state. This ends the recursive learning of *Q*.

The learning method is model free and does not require a working model of the environment but rather just the experience tuples $(s,a,r,s')$. The method also learns off-policy, that is, the learned policy is different from the performed policy. Therefore, an optimal policy $\pi ^*$ which provides the optimal action choice $a^* = \pi ^*(s)$ can be used to provide demonstrations through off-policy exploration to guide the agent to reward dense areas in the search space. We investigate two methods for utilizing demonstrations in deep reinforcement learning. The first is to simply initialize the *Q* network with weights learned from supervised learning with a dataset of demonstrations. Supervised learning is conducted as in Sect. 3.1 on a network with the same architecture as the *Q* network. The last layer uses a linear activation function instead of the softmax function used for classification in Sect. 3.1 as the *Q* network predicts continuous rewards for each available action. The agent uses random actions and its current policy to explore the environment, so initializing the network helps the agent explore behaviors similar to the teacher’s. The second approach is to use demonstrations from the optimal policy $\pi ^*$ to guide the agent’s exploration. The performance policy alternates between $a_t = \pi ^*(s_t)$ and random actions, to encourage exploration beyond the teacher’s demonstrations. Note that the choice between using demonstrations and random actions is performed once before each episode not before each action. It is easier in most applications for the teacher to provide demonstrations by performing the whole trajectory. This way the teacher is not required to produce an optimal action in the middle of the trajectory (such as in active learning techniques). The performance policy gradually shifts toward using the learned policy $\pi$ where $a_t={\mathrm{max}}_a Q(s_t,a;\pi )$, i.e., choose the action with the greatest predicted reward according to the trained neural network. In this approach, the information from demonstrations is independent of the agent’s learning process, while in the first approach the initialized policy changes with training.

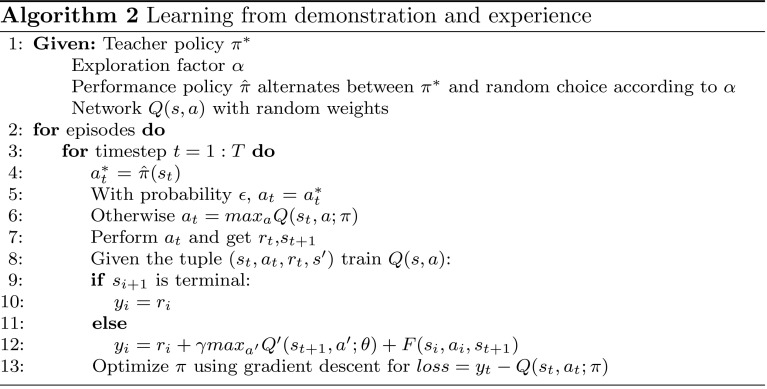



Algorithm 2 summarizes learning from experience using guiding demonstrations. The demonstrations are provided as in traditional learning by demonstration problems by simply performing the task in an optimal manner. Unlike [6], no specially designed labeled dataset is needed to pre-train $Q(s',a')$, which makes the training process more generic and streamlined. The task is assumed to be an MDP where the current state represents all past information (no extra context is needed to make a decision). Therefore, a single frame is used as the agent’s observation and the resulting policy is stationary (i.e., does not require information about the current position in the trajectory).

## Experiments

This section describes the experiments conducted to evaluate the methods detailed in Sect. 3. We present, discuss and analyze results comparing direct imitation learning, active learning and reinforcement learning methods. The experiments are conducted in the framework of MASH simulator [2] as well as a 2D Grid navigation task.

### Grid navigation task

**Fig. 2:**
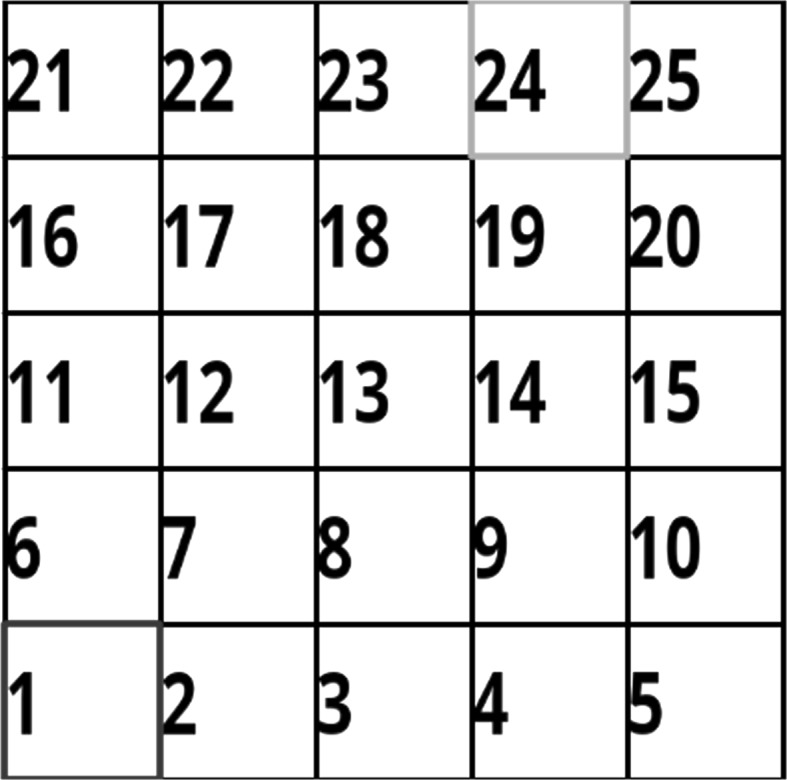
Illustration of the grid navigation task

This task is a simplified representation of navigation tasks which facilitates testing and analysis of learning algorithms in controlled manner. The environment is constructed of a grid where each cell is a state in the MDP and the agent is allowed to move between cells using 4 actions (Go Left, Go Right, Go forward, Go Back). Each state is represented by an $84 \times 84$ image of the number which reflect the number of this cell in the grid. These states are automatically generated given the dimension of the grid in terms of cells. The goal of the agent is to reach a target cell on the grid. Grids of dimensions $5\times 5$, $15\times 15$ and $30\times 30$ are used in this paper. This task is simple in that the environment is static, i.e., performing the same trajectory results in the same outcome. Therefore, the task does not pose the challenges of generalization. Another simplified aspect is having finite well defined states. However, the task presents other features which are relevant to real navigation tasks. Namely that it requires learning from raw visual data and requires long trajectories of dependent actions to achieve the target. The environment offers no intermediate positive feedback, while the agent is performing the task and only supplies a positive terminal reward when the target is reached. This is challenging as in a $30 \times 30$ grid, the shortest path to reach a reward consists of 57 steps. To give perspective, in a photo-realistic 3D environment which is used to train deep reinforcement learning agents [27], the shortest path to reach the reward is typically less than 20 actions. Figure 2 illustrates this task on a grid of size $5\times 5$. The agent’s starting position is shown by the blue marker, while the target state is highlighted in green.

To train the imitation learning policy, a dataset of demonstrations is collected by having a deterministic optimal policy control the agent. Pairs of state and action are captured and added to the training set. The demonstrations are used to train a deep neural network in a supervised manner. To evaluate the deep reinforcement learning algorithms DQN and A3C, the agent explores the environment using trial and error and receives a positive reward (+ 1) if it reaches the target and a negative reward (− 1) if it selects an action that would take it out of the grid. In this case, the agent’s position is not changed. The algorithms are run for 1000 epochs, each epoch consisting of 2500 steps. A testing step is conducted after each epoch where the result is 1 if the agent reached the target within a step limit and 0 otherwise.

### MASH simulator

MASH simulator [2] is a framework for evaluation of vision-based learning methods. It contains a number of tasks and environments designed for navigation. For each task, success and failure criteria as set, as well as a reward function and a teaching policy which considers 3D information from the simulator. The experiments in this paper are evaluated on 4 tasks.

#### Reach the flag

The goal of this task is to reach a flag which is placed randomly in a room. The task is considered successful if the agent reaches the flag within an allocated time limit.

**Fig. 3:**
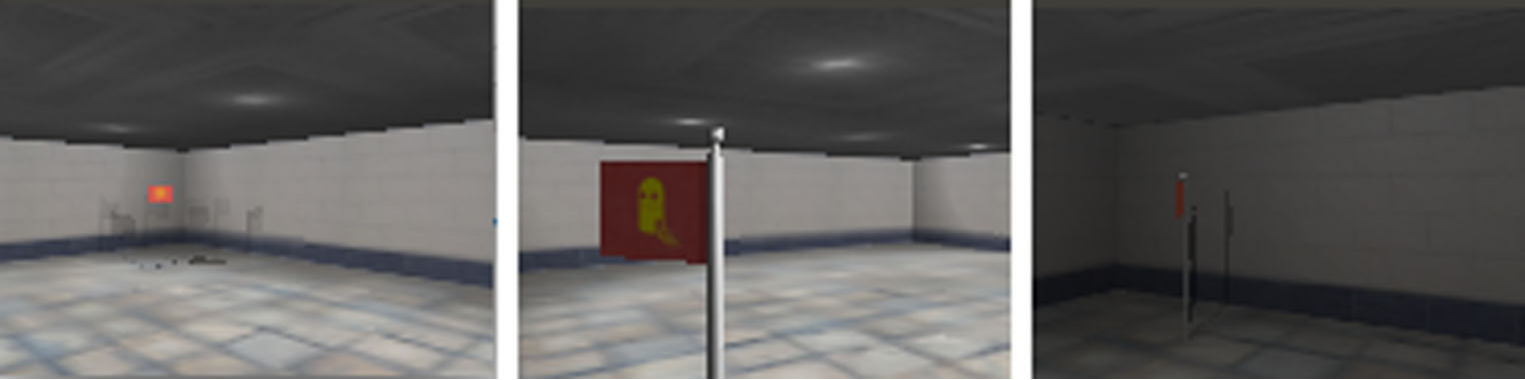
Sample images from “Reach the flag”

#### Follow the line

The goal of this task is to follow a pattern drawn on the floor which leads to the target. The pattern shows the direction in which the agent should move in order to reach the target. The task fails if the agent moves out of the patterned area.

**Fig. 4:**
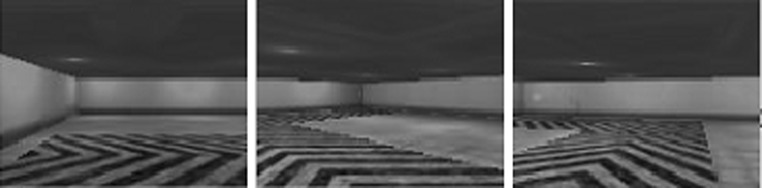
Sample images from “Follow the line”

#### Reach the correct object

The goal of this task is to reach an object while avoiding another similar looking object. The task fails if the wrong object is reached or if a time limit is exceeded before finding the correct object.

**Fig. 5:**
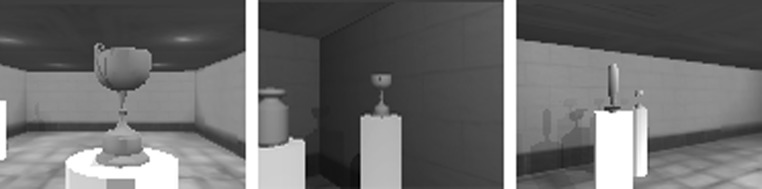
Sample images from “Reach the correct object”

#### Eat all disks

The objective of this task is to collect as many disks as possible within a time limit. Several black disks are laid out in a large room, and new disks appear once one is collected by the agent. Unlike other tasks, there are no failure criteria but only a score at the end of the given time.

**Fig. 6:**
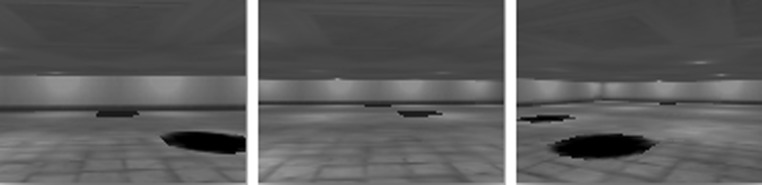
Sample images from “Eat all disks”

Figures 3, 4, 5, 6 show sample images of the 4 tasks. The images are shown in the same quality and size ($120 \times 90$) size produced by the simulator and used by the agent in the experiments.

For supervised learning, each task is trained on 20000 samples. Active learning is conducted using an active sample size of 5 and $10\%$ of the training data. Reinforcement learning algorithms are trained for 100 epochs of 250,000 steps each. A3C utilizes 8 parallel processes. And frame skipping of 5. Frame skipping can greatly help reinforcement learning by shortening the trajectory and enhancing exploration through taking bigger steps. However, delicate navigation can limit the number of frames to skip. For instance, in the “Follow the line” task, navigating the narrow corners of the patterned corridor fails when using high-frame skipping values even while following the optimal policy.

### Inter-process communication

For both simulators, the agent is decoupled from the simulator and the learning algorithm. This allows for generic independent modules and facilitates interchanging tasks and learning algorithms. A TCP connection is used to communicate between the different components.

**Fig. 7:**
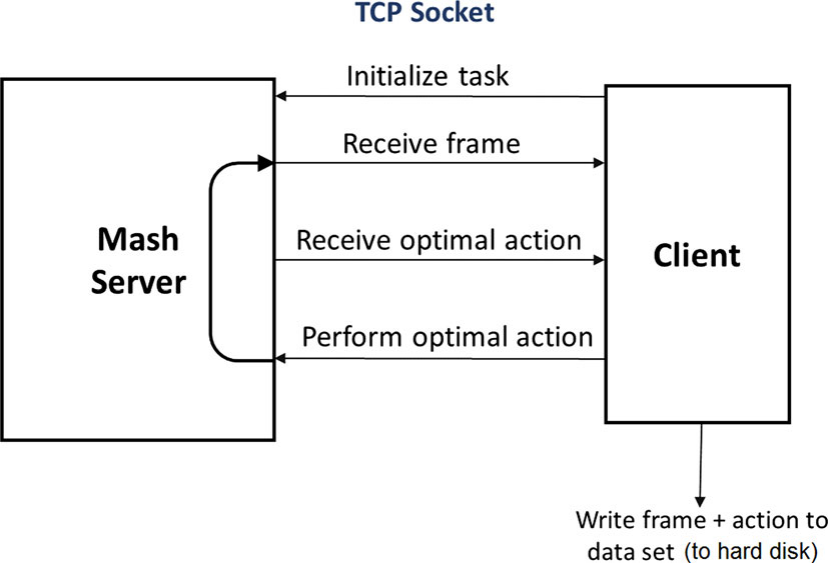
Dataset collection flowchart

**Fig. 8:**
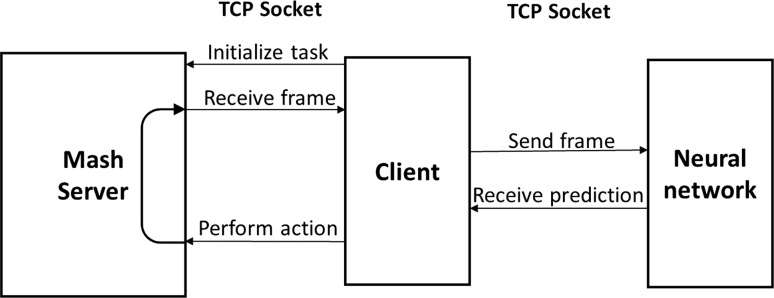
Imitation agent playing flowchart

**Fig. 9:**
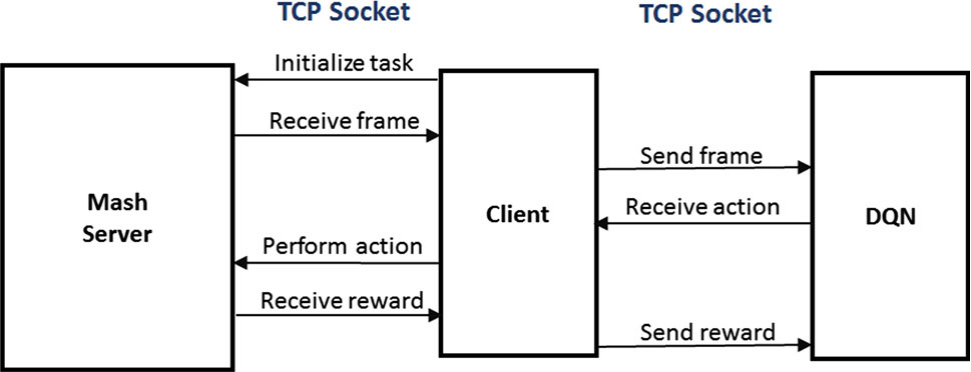
Reinforcement learning flowchart

Figure 7 shows the process of collecting demonstrations. The agent requests the current state from the simulator and receives an image and an optimal action. The agent saves the state action pair and sends the action back to the simulator for execution. The simulator updates the state, and the process is repeated. The collected dataset is used to train the neural network offline. Figure 8 shows the process of the agent performing a task based on the learned policy. Figure 9 presents the process of learning from experience used in combining reinforcement learning and imitation. The agent communicates with the simulator to receive the state of the environment and the reward and sends them to the learning network. The learning network uses this information to decide the next action and update the policy. The prediction action is sent to the agent which in turn communicates it to the simulator.

### Results

**Fig. 10:**
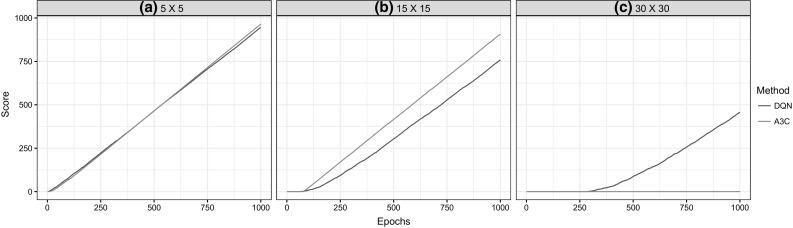
DQN and A3C results on the grid navigation task

Firstly, the results for experiments on the Grid task are presented. Since this task presents no states that are unseen in the demonstrations, for all grid sizes, the supervised policy was able to consistently solve the problem using only 5 demonstrations. Figure 10 shows results comparing DQN and A3C on the three grid sizes. Since success in this task is binary, the score counts how many epochs up to the current epoch have resulted in successful test sessions. This evaluation method produces a graph that shows the improvement and stability of the learned policy over training epochs.

The results on the Grid tasks show that considering static tasks, learning from demonstrations can be successful with far viewer training instances than learning from experience. Moreover, learning from experience becomes exponentially more difficult as the size of the grid increases. This is evident in the failure of A3C to learn on $30\times 30$ grid. This failure stems from the delayed rewards which makes obtaining feedback less frequent. The agent learns from the more readily available negative rewards to avoid the edges of the grid but is not able to reach the target.

Following, the results for experiments on the MASH simulator are presented. The same network and parameters are used to learn all tasks. The agent’s performance is evaluated by performing each task in the simulator for 1000 rounds. For the first 3 tasks, a success rate is calculated as the percentage of rounds in which the agent successfully completed the task out of 1000 rounds. For the fourth task “Eat all disks,” the evaluation measure used is the number of disks eaten in 1000 rounds. The results for deep reinforcement learning methods are reported after 100 epochs of training. For the imitation network, the classification error on an unseen test set is also reported. The test set consists of 20,000 samples collected from the teacher’s demonstrations.

**Fig. 11:**
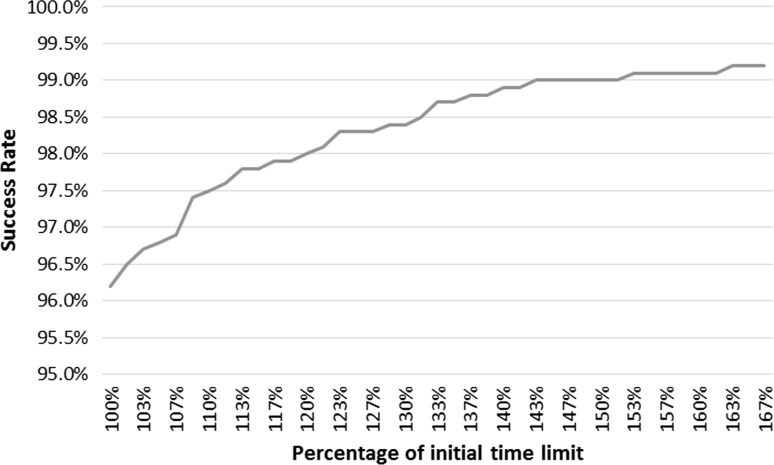
Results for “Reach the flag” task with increasing time limits

Table 3 shows the results for “Reach the flag,” “Reach the correct object” and “Follow the line.” The Success rates are reported for supervised learning, DQN and A3C as well test error for supervised learning. Supervised learning showed good performance on “Reach the flag” with a success rate of $96.2\%$. On “Follow the line” it resulted in a $40.7\%$ success rate. This is attributed to the failure criteria in “Follow the line” where a small deviation can result in the agent leaving the designated path and failing the round. While in “Reach the flag” the round is not failed unless the time limit is reached. If the agent makes an error in prediction and approaches the walls there is room for recovery. As the details of the walls become clearer the agent acts according to its learned policy and continues to search for the flag. Supervised learning resulted in a success rate of $53.1\%$ on “Reach the correct object.” Qualitative analysis shows that the agent fails to distinguish between the two objects and approaches them both resulting in a high failure rate. This could be attributed to the fact that the demonstrating policy does not avoid the wrong object if it stands between the agent and the target and only demonstrates avoiding the wrong object from a distance. Therefore, there are insufficient data to teach the agent to avoid the wrong object. The demonstrating policy performs the task with an $80.2\%$ success rate. A better demonstrator which actively avoids the wrong object in all cases could result in a better performance for the trained agent. This highlights direct imitation’s lack of generalization beyond the provided demonstrations. Both reinforcement methods failed to learn a robust policy to solve any of the 3 tasks. Qualitative analysis shows that all successful attempts during testing were achieved by chance without any clear pattern in the learned policy. Since “Follow the line” requires a longer trajectory and is not as fault tolerant as the other tasks, it is less suitable for random exploration. Thus, reaching the target by chance is more difficult and the success rate is $0\%$. The test errors for all 3 tasks are relatively low and don’t reflect the failure rates. This shows that small prediction errors can lead the agent to face situations that are not represented in the demonstrations and therefore propagate erroneous behavior. Since the agent in “Reach the flag” was able to correct its behavior following wrong prediction, we evaluate the effect of the time limit on the agent’s success. Figure 11 shows the success rate against different time limits represented as percentages of the original time limit. The graph shows that the success rate improves with longer time limits, which shows that continuing to follow the learned policy can result in success even after sub-optimal behavior.

**Table 3:** Direct imitation results

Task	Reach the flag (%)	Reach object (%)	Follow the line (%)
Direct imitation	96.20	53.10	40.70
DQN	6.40	6.00	0.00
A3C	7.60	8.9	0.00
Error	2.48	4.06	0.86

Table 4 shows results for “Eat all disks.” The table compares the scores achieved by direct imitation, DQN, A3C and the optimal policy. The results show that direct imitation achieves $97.9\%$ of the score achieved by the optimal policy while again learning from experience failed to produce an effective policy.

**Table 4:** “Eat all disks” results

Task	Direct imitation	Optimal policy	DQN	A3C
Score	1051	1073	51	45
Error	1.70%	–	–	–

**Fig. 12:**
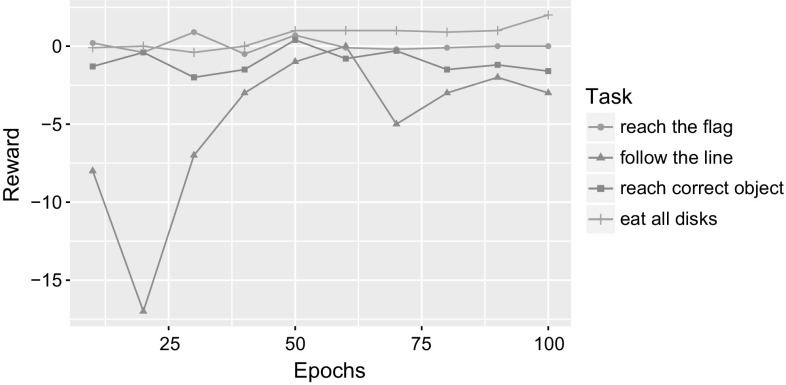
Results for DQN on navigation tasks in MASH simulator

**Fig. 13:**
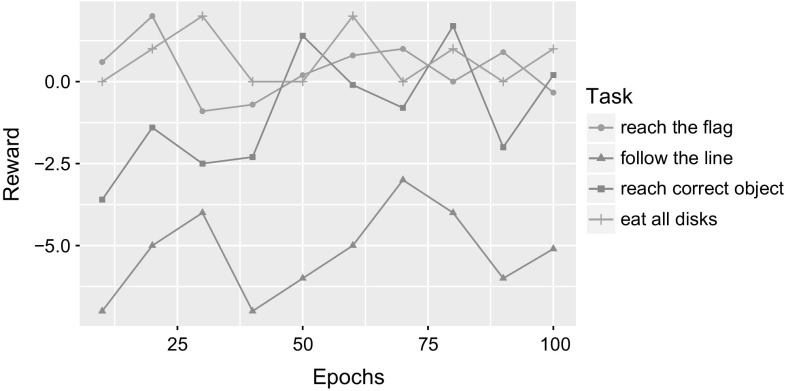
Results for A3C on navigation tasks in MASH simulator

Figures 12 and 13 show results for the 4 tasks in terms of rewards received for DQN and A3C, respectively, over 100 epochs. The test results are reported every 10 epochs and show rewards averaged over the test rounds. The graphs show no pattern of improving the performance with the increasing number of epochs.

**Fig. 14:**
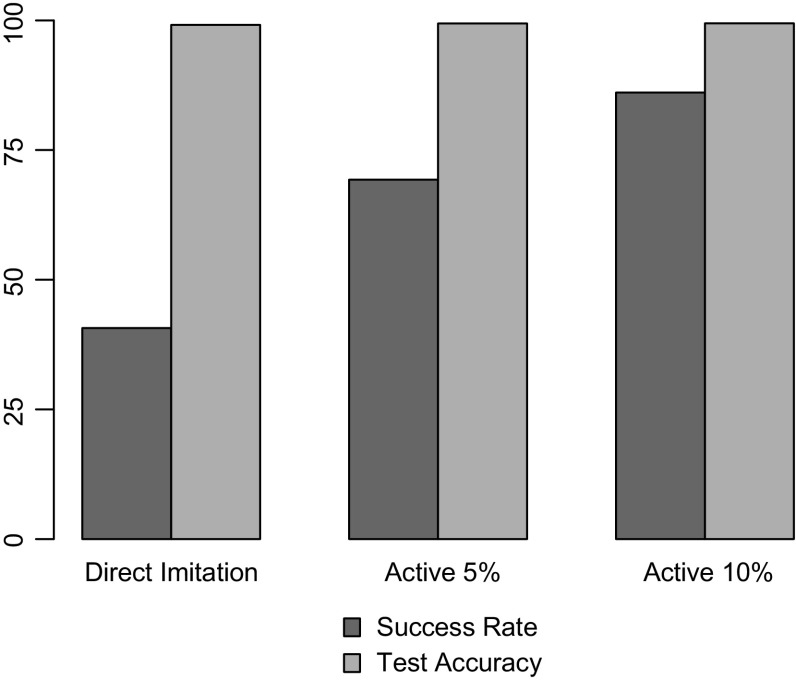
Results for active learning on “Follow the line” task

In Fig. 14, the proposed active learning method is evaluated on “Follow the line.” Active learning is not used on the other tasks as the demonstrating policies keep track of the target’s location even if it not in the current frame. This contradicts with the approach of learning solely from the current visual data and requires either incorporating memory in the learning process or replacing the policy that provides active samples. The graph compares the success rate and test error of direct imitation against those of active learning using $5\%$ and $10\%$ of the training data. The results show that active learning significantly improves the success rate of the agent. Increasing the size of the active dataset is shown to further improve the performance. Comparing the improvement in classification error against that in success rate emphasizes the point that poor agent behavior stems from situations that are not represented in the teacher’s demonstrations.

**Fig. 15:**
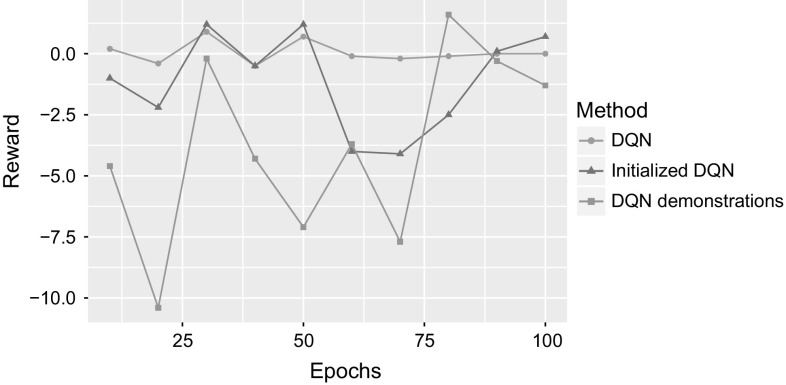
Results for combining learning from demonstrations and experience on “Reach the flag”

Next we evaluate combining learning from demonstrations and experience. The two methods proposed in Sect. 3.2 to help DQN using demonstrations are compared to traditional DQN on the “Reach the flag” task. “Initialized DQN” initializes the policy network of DQN with the parameters learned from supervised learning, while “DQN demonstrations” refers to using demonstrations from the optimal policy to perform off-policy rollouts. Figure 15 shows the average rewards every 10 epochs for 100 epoch. The graph shows that utilizing demonstrations using the two proposed methods did not enhance the performance of DQN. The initial policy learned from demonstrations is quickly overwritten and thus provides no benefit to the learning policy or the rollout policy. This happens as there are no constraints to preserve the initial policy once DQN training starts. Guiding the agent by utilizing demonstrations in exploration also did not show any improvement. By looking at the probability distribution of the output layer of the network, we attribute this failure to the fact that the cost function used in DQN training does not consider output nodes other than the performed action. Therefore, when applying a rollout policy of optimal actions, the probabilities of non-used actions change arbitrarily. A cost function that includes all actions could be considered, but since DQN uses a periodically updated target network, the learned parameters for the performed actions will be overwritten with every update.

Overall, the results of the proposed learning from demonstrations method show good performance on 3 out of the 4 tasks. They demonstrate the effectiveness of active learning to significantly improve a weak policy with a limited number of samples. Even without active learning the agent can learn a robust policy for simple navigation tasks. Comparisons with deep reinforcement learning methods show that learning from demonstrations can learn the same task with substantially fewer training instances. Results of deep reinforcement learning methods showed that learning becomes more difficult with longer trajectories and that they failed to learn the 4 tasks on MASH simulator.

## Conclusion and future directions

In this paper, we propose a framework for learning autonomous policies for navigation tasks from demonstrations. A generic learning process is employed to learn from raw visual data without integrating any knowledge of the task. This method is compared to two state-of-the-art deep reinforcement learning methods. Active learning is employed to help the agent generalize to unseen situations. Methods for combining learning from demonstrations and experience are also investigated to improve the generalization ability of the agent while taking advantage of provided demonstrations. The experiments are conducted on a testbed that facilitates reproduction, comparison and extension of this work. The results show that CNNs can learn meaningful features from raw images of 3D environments and learn a policy from demonstrations. They also show that active learning can significantly improve a learned policy with a limited number of samples. Moreover, it is shown that learning from demonstrations can be successful with significantly fewer instances than learning from experience and outperforms deep reinforcement learning methods on the 4 3D navigation tasks used. The comparison between learning from demonstrations and experience highlights the limitations of both techniques. Direct imitation can generalize poorly if no appropriate active samples are available. While learning by trial and error from scratch can be ineffective in tasks with long trajectories and sparse rewards.

In the future we aim to further investigate tackling the generalization problem in imitation learning methods. More general active learning methods are to be investigated in order to work with a larger variety of tasks. Incorporating memory of past actions in imitation learning would allow for active learning with different expert policies. Although initial results were not successful, integrating learning with experience and demonstrations can help with generalization without requiring teacher involvement. In the next step we aim to investigate using guiding demonstrations with reinforcement learning methods that use different cost functions and do not require target networks. Furthermore, adapting the online learning methods in [44] can speed up retraining while overcoming the catastrophic forgetting phenomenon. This can also potentially allow one network to learn multiple tasks.
